# Inference of synergy/antagonism between anticancer drugs from the pooled analysis of clinical trials

**DOI:** 10.1186/1471-2288-13-77

**Published:** 2013-06-12

**Authors:** Wenfeng Kang, Robert S DiPaola, Alexei Vazquez

**Affiliations:** 1 Department of Radiation Oncology and Center for Systems Biology, The Cancer Institute of New Jersey, 195 Little Albany St, New Brunswick, NJ 08901, USA; 2 Division of Medical Oncology, The Cancer Institute of New Jersey, University of Medicine and Dentistry of New Jersey - Robert Wood-Johnson Medical School, New Brunswick, NJ, USA

**Keywords:** Cancer therapy, Clinical trial, Overall response rate, Combinatorial therapies, Clinical synergy, Systems biology

## Abstract

**Background:**

Drug interactions can have a significant impact on the response to combinatorial therapy for anticancer treatment. In some instances these interactions can be anticipated based on pre-clinical models. However, the anticipation of drug interactions in the clinical context is in general a challenging task.

**Methods:**

Here we propose the pooled analysis of clinical trials as a mean to investigate drug interactions in anticancer therapy. To this end we collected 1,163 Phase II clinical trials with response data on over 53,745 subjects.

**Results:**

We provide statistical definitions of drugs resulting in clinical synergy and antagonism and identify drug combinations in each group. We also quantify the possibility of inferring interactions between three or more drugs from parameters characterizing the action of single and two-drugs combinations.

**Conclusions:**

Our analysis provides a statistical methodology to track the performance of drug combinations in anticancer therapy and to quantify drug interactions in the clinical context.

## Background

Given the low rate of phase III trials success, complimentary options to optimize earlier trial development, especially with combination therapy, would be critically important [1]. The identification of effective drug combinations is, however, a challenging task [2], given the large number of potential targets and agents available or under investigation. For example, 100 FDA approved drugs would result in about 5,000 two-drug combinations, and the number increases exponentially if we consider combinations of multiple agents. Additionally, preclinical drug combination screens explore various concentration ranges for each drug in a combination, making the exhaustive screen of all possible drug combinations difficult. Although preclinical studies historically have helped inform early trial development of combination therapies, the contributory value of phase II trials and ultimate success in identifying agents with a survival impact in phase III trials has been reported to be low, warranting new methodologies [3].

Meta-analyses pooling together many clinical trials have been used to better quantify the performance of a new anticancer treatment (single agents or combinations) relative to a standard treatment [4-7]. We hypothesized that data analyzed across large numbers of clinical trials could also be utilized to obtain an estimate of the interaction between anticancer drugs. Several drug combinations have been tested in clinical trials in the past years, providing a unique resource to understand the response patterns of drug combinations. A typical measure for treatment success is the clinical overall response rate (ORR), defined as the percentage of patients whose cancer shrinks (partial response) or disappears (complete response) after treatment. By observing successful, and unsuccessful, combinations defined by ORR in multiple cancer types we hypothesized that we could identify synergistic drug combinations with a response rate higher than what is expected. In addition to increased response rate, we hypothesized that we could identify drug interactions with antagonistic effects. About 20-30% of all adverse reactions to drugs are caused by interactions between drugs [8], underscoring the need to identify antagonistic drug interactions as well.

Although theoretical foundations for the experimental design to *in vitro* study drug combinations are well established [9], methodologies to assess large and varied clinical datasets are limited. In this work, we develop statistical methodologies to characterize drug interactions directly from clinical data. Specifically, we study the response rates of single drug and drug combinations tested in Phase II clinical trials for their anticancer activity. Our main goal is to uncover general patterns that could inform future approaches aiming to identify effective drug combinations for anticancer treatment.

## Methods

### Study design

To test our statistical methodology, on May 7, 2010, we searched PubMed with the following search key: cancer phase II clinical trial overall response rate. From the list of returned abstracts we selected in order of appearance the first 1,000 clinical trials. This number was chosen to balance the effort of manually extracting the required data from the PubMed abstracts and the desire to include as many trials as possible. Following an initial assessment of our methodology with that subset of clinical trials, on August 9, 2011, we searched PubMed again to extract new reports between this date and the previous search. This resulted 163 additional trials adding to a total of 1,163 trials. The reason for the latter search was to allow us to investigate more recent trends. We did not found any significant differences from the analysis of the initial set of 1,000 trials and the final set of 1,163 trials. The complete list of trials is reported in the Additional file 1. Our primary measure for treatment success was the clinical overall response rate (ORR), defined as the percentage of patients whose cancer shrinks (partial response) or disappears (complete response) after treatment. Recognizing the limitations of comparing response rates for each cancer type across separate trials, we chose the overall response rate as the main outcome measure. This choice was based on the assumption that most phase II trials used standard RECIST response criteria, and were powered for a clinically relevant response rate that could lead to a “go no-go” decision for a phase III study. Overall, 184 agents were tested as single agents or in combination in the collected trials.

### Observed ORR

Each clinical trial reports the number of patients with an overall response (*n*) and the trial sample size (*N*). For a given combination tested in a given trial, *n* is modeled as a random variable following the binomial distribution $P\left(n|N,p\right)=\left(\begin{array}{l} N\\ n \end{array}\right)p^{n}{\left(1-p\right)}^{N-n}$, where *p* is an unknown parameter representing the probability that a patient manifest a partial or complete response to the treatment. The Bayesian posterior distribution of *p* is given by a beta distribution $P\left(p|n,N\right)=\frac{1}{B\left(\alpha ,\beta \right)}p^{\alpha -1}{\left(1-p\right)}^{\beta -1}$, where *α*=*n* and *β*=*N*-*n* and *B*(*α*,*β*) is the beta function (Additional file 2). For a given combination tested in multiple trials, we take into account that *p* may be different due to the use of different dose, schedule or cancer subtype on each trial. To account for these differences, we constructed a statistical methodology that models the existence of multiple classes of trials (among those testing the same combination) with different values of *p* (Additional file 2). In our dataset, there were 166 combinations tested in two or more trials. When pooling the clinical trials by the combination tested, in 142 of these combinations the data indicates that all trials are statistically equivalent as determined by the Bayesian method. In these cases we pooled together the data from clinical trials testing the same combination even though some were conducted in different cancer subtypes. For the remaining 24 combinations, there are significantly different response rates depending on the cancer type. In this latter case the Bayesian method returns two or more groups, each containing one or more cancer types. When each group was represented by only one trial we removed those trials from our search of synergistic/antagonistic combinations. Otherwise we removed the trials in the group with lowest number of trials. The excluded trials are indicated in the Additional file 3. These trials were removed because the reported ORR was inconsistent with the report by trials testing the same combination in the same cancer type. When all trials are statistically equivalent, *p* follows a beta distribution with *α* = ∑_*i*_*n*_*i*_ and *β* = ∑_*i*_(*N*_*i*_ − *n*_*i*_), where the index *i* runs over all trials testing the combination. The expected probability of response rates is computed from the mean of the beta distribution mean(*p*) = *α*/(*α* + *β*) = ∑_*i*_*n*_*i*_/∑_*i*_*N*_*i*_, and the associated ORR is computed as ORR_0_ = 100%×mean(*p*).

### Null model for combinations of two non-interacting agents

In the absence of agent interactions, the probability that a patient responds to a treatment based on two agents equals one minus the probability that he/she does not respond to either treatment: *q*_*ij*_ = 1 − (1 − *p*_*i*_)(1 − *p*_*j*_), where *p*_*i*_ and *p*_*j*_ are the response probabilities for each agent when used as a single agent. The probabilities *p*_*i*_ were estimated using trials where the agents were tested as single agents. In a trial where *N* patients were treated with the two agents *i* and *j*, we expected *n* responses with a binomial probability distribution $P\left(n|N,q_{\mathrm{ij}}\right)=\left(\begin{array}{c} N\\ n \end{array}\right)q_{\mathrm{ij}}^{n}{\left(1-q_{\mathrm{ij}}\right)}^{N-n}$. We estimated the probability that there was synergy as the probability to obtain as many or more responses given a non-interacting agents hypothesis: $p_{\mathrm{synergy},\mathrm{ij}}=\sum_{m=n}^{N}P\left(m|q_{\mathrm{ij}},N\right)$. Similarly, we estimated the probability that there was antagonism as the probability to obtain as many or less responses, given a non-interacting hypothesis: $p_{\mathrm{antagonism},\mathrm{ij}}=\sum_{m=0}^{n}P\left(m|q_{\mathrm{ij}},N\right)$. Finally, the expected ORR under the assumption of non-interacting agents was defined as ORR_1,*ij*_ = 100*%*mean(*q*_*ij*_) = 100*%*[1 − (1 − mean(*p*_*i*_))(1 − mean(*p*_*j*_))]. The simulations to test the null (non-interacting) model approach were performed as follows. Given a sample size *N*, uniformingly sampled values between 0 and 1 were generated for the probability of response to drug 1, *p*_1_, the probability of response to drug 2, *p*_2_, and the probability to respond to the combination of drug 1 and 2, *p*_12_. Using the probabilities *p*_1_ and *p*_2_, the probability of response to the combination of drug 1 and 2 in the absence of drug interactions, *q*_12_ = 1 − (1 − *p*_1_)(1 − *p*_2_), was computed. Then, *n*_1_, *n*_2_ and *n*_12_ responses out of *N* attempts were generated using a binomial model with probability of success *p*_1_, *p*_2_ and *p*_12_, respectively. Using the generated number of responses, the posterior means *p*_0,1_ = *n*_1_/*N*, *p*_0,2_ = *n*_2_/*N* and *p*_0,12_ = *n*_12_/*N* were computed. Finally, using *q*_0,12_ = 1 − (1 − *p*_0,1_)(1 − *p*_0,2_) and *n*_12_ as input, *p*_*synergy*,12_ and *p*_*antagonism*,12_ were computed. This procedure was repeated 10,000,000 times. The manifestation of synergy/antagonism was determined by comparing the expected ORR as obtained from the null model ORR_1_ = 100%*q*_12_ with the ORR as observed ORR_0_ = 100%*p*_12_. Specifically, the probability for synergy *p*_*synergy*,12_ was calculated as the fraction of times that ORR_0_>ORR_1_ and *p*_*synergy*,12_≥0.05. Similarly, the probability for antagonism *p*_*synergy*,12_ was calculated as the fraction of times that ORR_0_<ORR_1_ and *p*_*antagonism*,12_≥0.05. To compute these fractions we divided the [0–1] interval in bins of size 0.01.

### Two-agents approximation to the ORR

In the 2-agent approximation model the ORR for each agent combination *c* is derived from parameters quantifying the response to a single agent and the interaction between two agents. These combinations are constructed out of *N*_*a*_ agents. The combinations are specified using the agent to combination matrix_*.*_*s*_*ci*_, where *s*_*ci*_ = 1 if agent *i* is part of the combination *c* and *s*_*ci*_ = 0 otherwise. The parameter *h*_*i*_ denotes the response rate to agent *i* when tested as a single agent. The parameters *J*_*ij*_ are introduced to quantify corrections due to agent interactions. The probability *p*_*c*_ to respond to a combination *c* is written as $p_{c}=\sum_{i=1}^{N_{a}}s_{\mathrm{ci}}h_{i}+\sum_{i=1}^{N_{a}-1}\sum_{j=i+1}^{N_{a}}s_{\mathrm{ci}}s_{\mathrm{cj}}J_{\mathrm{ij}}$ and the associated overall response rate is ORR_*c*_ = 100%×*p*_*c*_. To understand the equation for *p*_*c*_, it is better to focus on the case when the drugs do not interact. In this case $p_{c}=1-\prod_{i=1}^{N_{a}}{\left(1-h_{i}\right)}^{s_{\mathrm{ci}}}=\sum_{i=1}^{N_{a}}s_{\mathrm{ci}}h_{i}-\sum_{i=1}^{N_{a}-1}\sum_{j=i+1}^{N_{a}}s_{\mathrm{ci}}s_{\mathrm{cj}}h_{i}h_{j}$. Therefore, in the absence of interactions the equation of *p*_*c*_ can be written as we postulated above with $J_{\mathrm{ij}}^{0}=-h_{i}h_{j}$. The difference between an observed *J*_*ij*_ and the non-interacting value $\Delta J_{\mathrm{ij}}=J_{\mathrm{ij}}-J_{\mathrm{ij}}^{0}$ is used to quantify synergy (when positive) and antagonism (when negative). The 2-agent model can be rewritten as $p_{c}=\sum_{m=1}^{N_{v}}A_{\mathrm{cm}}x_{m}$, where *N*_*v*_ is the number of variables (unknown *hs* and *Js*), the *x*_*m*_ are variables representing the *hs* and *Js* parameters, and *A*_*cm*_ is the variable to a combination matrix (*A*_*cm*_ = 1 if variable *m* appears in the equation for combination *c* and *A*_*cm*_ = 0 otherwise). The variables *x*_*m*_ were estimated by solving the corresponding least-squares problem, with some additional considerations to account for the variability in the empirical estimates of *p*_*c*_ (Additional file 2). The 2-agent approximation to the ORR was given by ORR_2,*ij*_ = 100*%*[∑_*i*_*s*_*ci*_mean(*h*_*i*_) + ∑  _*i* <*j*_*s*_*ci*_*s*_*cj*_*mean*(*J*_*ij*_)].

### Kolmogorov-smirnov test

The kolmogorov-smirnov test [10] was used to determine the probability that two lists of ORRs were generated from the same distribution.

## Results and discussion

### Statistical equivalence of trials testing the same combination

The response rate to a combination of agents may depend on the dose of each agent, the treatment schedule, and the cancer subtype. We developed a statistical methodology that determines if a set of trials testing the same combination is statistically equivalent, or if there are trials with statistically different response rates. To this end, we developed a Bayesian method that takes into account the variations in the estimated response rates due to finite sample sizes. This Bayesian method determines whether the number of responses reported in a set of clinical trials are consistent with a unique response rate, up to variations determined by the finite sample sizes, or whether the trials cluster in two or more groups with statistically significant response rates. In our dataset, there were 166 agent combinations that were tested by two or more clinical trials. In 142 combinations the observed response rates were statistically equivalent. Only in 24 combinations there is evidence of trials with statistically different response rates (Additional file 3).

We note that the observation of statistical equivalence does not imply that the response rates do not depend on dose or cancer type. By necessity, we made the assumption that in most trials within the dataset studied, the agent doses are standard based on standard maximal tolerated doses defined by prior phase I trials. Response rates were also comparable because similar agent combinations or combinations of similar drug class were usually tested in the same cancer subtype. We recognized that targeted therapies were often used to treat patients selected on the basis of a companion biomarker. For example, within the studied dataset, there were 32 trials testing trastuzumab as a single agent or in combination. In all cases the patients enrolled were Her2/neu+, the target of trastuzumab. Although we assumed that within the studied dataset the trials associated with the same agent combination are statistically equivalent, we acknowledged that in some instances the results that follow are biased towards specific cancer subtypes. For example, all the interactions with trastuzumab are relevant in the context of Her2/neu+patients and interactions with radiation are relevant in the context of localized disease. These biases will be reported when necessary.

### Trends as a function of the number of agents combined

**Figure 1:**
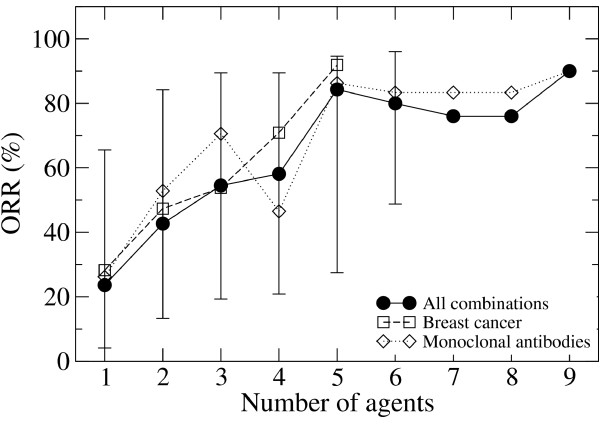
**The ORR increases with increasing the number of agents in the treatment.** The ORR as a function of the number of agents in the combination. The symbols represent the median across all trials testing that many agents and the bars the 90% confidence intervals. The 90% confidence intervals for the combinations tested in breast cancer and those using monoclonal antibodies have been omitted to avoid confusion.

The Bayesian method also reports the posterior mean of the response rate associated with every single agent and combination in our dataset. To investigate trends as a function of the number of agents tested, we stratified the combinations by the number of agents tested. For a given number of agents, we collected the posterior mean response rates of all combinations testing that many agents. From this list we determined the median and the lower and upper bound for the 90% confidence interval, as the combination with posterior response rate higher/lower than 50%/50%, 5%/95% and 95%/5% of the samples, respectively. We observed that the median over the posterior mean ORRs increased with increasing the number of agents in the combination (Figure 1). For single agents, the median ORR was below 30% and increased to above 70% for treatments with five or more agents. Similar trends were observed when restricting the analysis to trials in breast cancer (171 clinical trials), indicating that these observations hold for specific cancer subtypes as well. The ORR 90% confidence interval for combinations testing a given number of agents was quite broad. Therefore, not all combinations testing more agents outperformed those testing lesser agents.

### Targeted vs non-targeted therapies

Among the 514 combinations tested, 85 included novel monoclonal antibodies, allowing us to test whether targeted therapies are improving response rates. As expected, based on prior randomized trials, the distribution of posterior mean ORRs for trials testing monoclonal antibodies was shifted toward higher ORRs relative to the distribution for trials not including monoclonal antibodies (54% vs 46%, p-value of 0.0042 based on a Kolmogorov-Smirnov test). Furthermore, the median over posterior ORRs was higher for combinations including monoclonal antibodies independently of the number of agents in the combination (Figure 1), with the exception of combinations with four agents.

### Clinical synergy

We then focused on agent combinations resulting in trials with ORRs that were significantly different than what expected from a null model with no interactions between the agents tested. From the clinical point of view, a combination was deemed *synergistic* when its ORR was significantly higher than what was expected from the null model of non-interacting agents, and *antagonistic* when significantly lower. The null model for non-interacting drug combinations is mathematically described in the methods section. In simple words, in the absence of drug interactions the probability that a patient responds to a two-drug combination is equal to the probability that he/she responds to at least one drug in the combination. The probability that a patient responds to at least one drug in the combination can be estimated using as input the trials testing the drugs as single agents. Once we have an estimate of the probability that a patient responds to the two-drug combination in the absence of interactions, we can determine the probability to observe as many or more responses in a trial with a given sample size (*p*_*synergy*_), the probability to observe as many or less responses (*p*_*antagonism*_) and the expected response rate in the absence of drug interactions ORR_1_.

**Figure 2:**
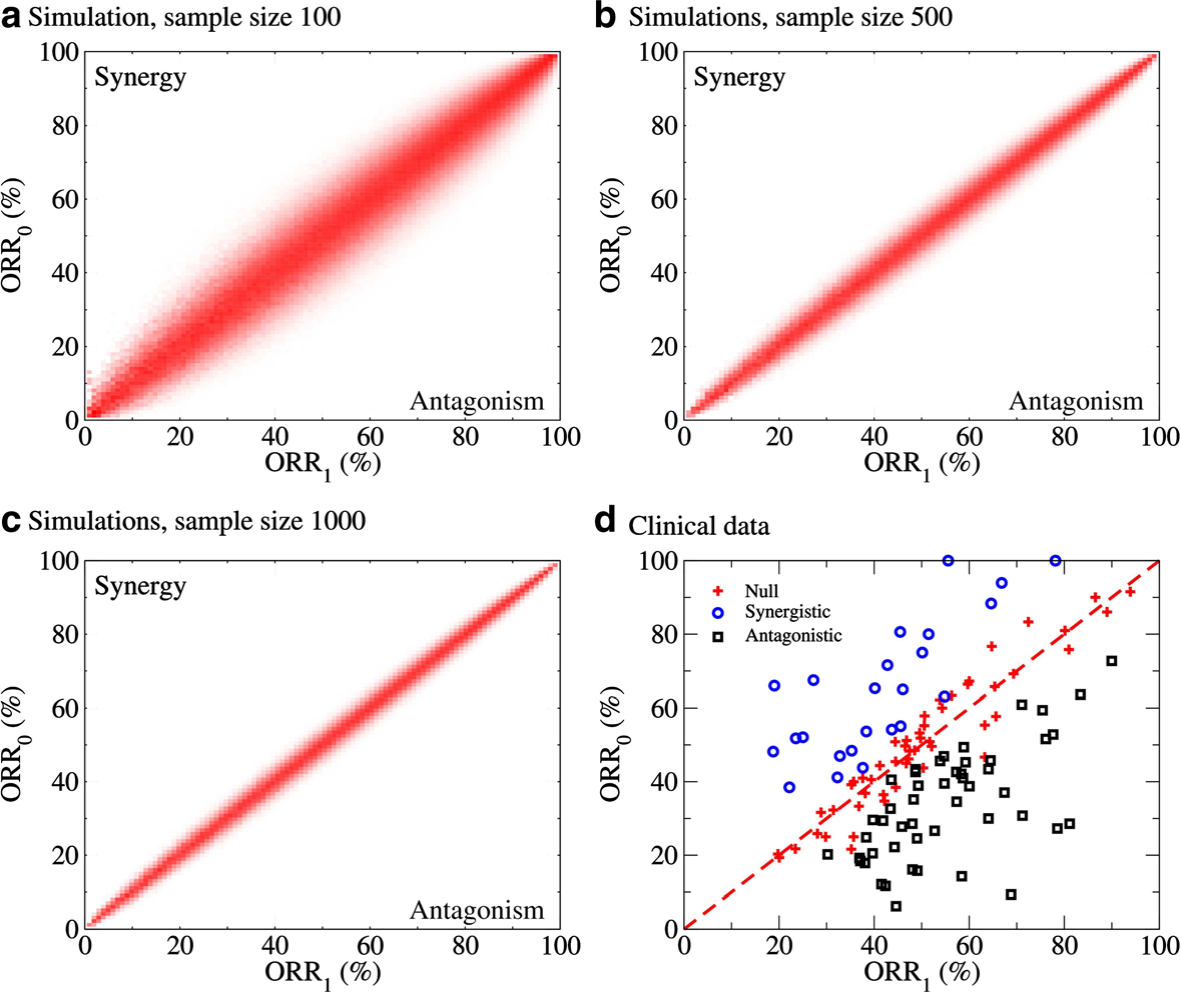
**Clinical synergy and antagosnism.** The observed ORRs (ORR_0_) as a function of the expected ORR assuming there are no agent-agent interactions (ORR_1_). Panels **a**)-**c**) show results obtained from the simulation of clinical trials with the reported sample sizes (see Methods). The red color intensity is proportional to fraction of times that a two-agent combination with the corresponding observed ORR_0_ and expected ORR_1_ is dimmed non-interacting. **d**) The results obtained for the two-agent combinations in the clinical data. The diagonal line represents the perfect agreement between the two. Circles represent combinations with evidence for synergy (*p*_*synergy*_ < 0.05), squares represent combinations with evidence for antagonism (*p*_*antagonism*_ < 0.05), and the pluses represent combinations without evidence for synergy or antagonism (*p*_*synergy*_≥0.05 and *p*_*antagonism*_≥0.05).

The ability of this approach to uncover synergistic/antagonistic interactions will depend on the sample sizes and the degree of synergy/antagonism. This is illustrated in Figure 2a-c for simulated data with sample sizes 100, 500 and 1,000. The red areas highlight the region where the number of observed responses is consistent with the null model of non-interacting agent. In turn, the white areas represent cases where our approach correctly determines that the two-drug combination is either synergistic or antagonistic. As expected, our ability to discriminate from the null model increases as the sample size increases. Of course, in addition to the statistical significance for synergy/antagonism we should focus on the effect size, i.e. how much the observed response rate (ORR_0_) deviates from what expected from the null model for non-interacting drugs (ORR_1_).

Figure 2d shows the classification of the two-agent combinations in our dataset into synergistic (*p*_*synergy*_ < 0.05), antagonistic (*p*_*antagonism*_ < 0.05) or non-interacting (*p*_*synergy*_≥0.05 and *p*_*antagonism*_≥0.05). Table 1 and 2 report the two-agent combinations with evidence for synergy and antagonism, respectively. About half of the predicted synergistic combinations are the standard of care in specific cancer types (e.g., docetaxel+doxorubicin in breast cancer), indicating that our analysis captures the current trends in cancer treatment (Table 1). The remaining synergistic combinations should be further studied to evaluate their potential to improve cancer treatment. In contrast, only one antagonistic drug combinations is currently used as standard of care for the corresponding cancer subtypes (Table 2).

**Table 1:** **Synergistic 2**-**agent combinations**

**Agent 1**	**Agent 2**	Standard of care*	**Expected *ORR***_**1**_	**Observed *ORR***_**0**_	***p***_***synergy***_	**Cancer subtype**
Docetaxel	Doxorubicin	Yes	40	65	7.70E-43	Breast cancer
Oxaliplatin	5-fluorouracil	Yes	25	52	1.82E-17	Colorectal, gastric cancer
Irinotecan	Etoposide	No	19	66	4.98E-13	Lung cancer
Doxorubicin	Ifosfamide	Yes	19	48	1.20E-09	Gynecologic, soft tissue sarcoma
Bortezomib	Thalidomide	Yes	56	100	2.13E-09	Myeloma
Capecitabine	Irinotecan	No	35	48	9.53E-09	Colorectal, gastric, lung cancer
S-1	Irinotecan	No	33	47	1.20E-07	Colorectal, gastric, lung cancer
Oxaliplatin	Doxorubicin	No	27	68	1.30E-07	Ovarian cancer
Oxaliplatin	Irinotecan	Yes	24	52	4.30E-06	Colorectal, lung cancer
Capecitabine	Radiation	No	65	88	3.11E-05	Cervical cancer
Rituximab	Alpha-interferon	No	67	94	2.48E-04	Lymphoma
Oxaliplatin	Capecitabine	Yes	38	44	3.41E-03	Colorectal, gastric cancer
5-fluorouracil	Irinotecan	Yes	22	38	5.87E-03	Colorectal cancer
Capecitabine	Alpha-interferon	No	38	54	3.34E-02	Renal cancer
Fludarabine	Thalidomide	No	78	100	4.05E-02	Leukemia

*By National Comprehensive Cancer Network (NCCN) guidelines.

The combinations with an observed ORR (ORR_0_) that is significantly higher than the expected ORR in the absence of agent interactions (ORR_1_).

**Table 2:** **Antagonistic 2**-**agents combinations**

**Agent 1**	**Agent 2**	Standard of care*	**Expected *ORR***_**1**_	**Observed *ORR***_**2**_	***p***_***antagonism***_	**Cancer subtype**
Docetaxel	Topotecan	No	42	12	2.94E-11	Lung, ovarian cancer
Bortezomib	Rituximab	No	78	53	1.20E-09	Lymphoma
Temozolomide	Alpha-interferon	No	38	18	5.87E-08	Melanoma
Capecitabine	Bortezomib	No	58	14	9.02E-08	Breast cancer
Fludarabine	Alemtuzumab	No	83	64	1.27E-07	CLL, leukemia
Bevacizumab	Temozolomide	No	48	16	1.41E-07	Melanoma
Capecitabine	Trimetrexate	No	45	6	2.15E-06	Colorectal cancer
Docetaxel	Irinotecan	No	37	18	1.15E-05	Gastric, lung, ovarian cancer
Bortezomib	Temsirolimus	No	64	30	1.25E-05	Myeloma
Temozolomide	Interleukin-2	No	49	16	1.97E-05	Melanoma
Docetaxel	Gefitinib	No	71	31	2.29E-05	Lung cancer
Docetaxel	5-fluorouracil	No	38	25	1.71E-04	Gastric, head and neck cancer
Rituximab	Temsirolimus	No	75	59	2.44E-03	Lymphoma
Temozolomide	Radiation	No	64	46	2.51E-03	Breast cancer
Fludarabine	Alpha-interferon	No	76	52	2.55E-03	Lymphoma
G-CSF	Rituximab	No	71	61	4.69E-03	CLL, lymphoma
Doxorubicin	Bevacizumab	No	40	21	9.11E-03	Breast cancer
Docetaxel	Imatinib	No	46	28	2.09E-02	Breast cancer
Docetaxel	Capecitabine	Yes	49	43	4.48E-02	Breast, gastric cancer
Vinorelbine	Mitoxantrone	No	42	29	4.62E-02	Breast cancer

*By National Comprehensive Cancer Network (NCCN) guidelines.

The combinations with an observed ORR (ORR_0_) that is significantly lower than the expected ORR in the absence of agent interactions (ORR_1_).

We tested the hypothesis that synergy was more common in combinations using monoclonal antibodies, a class of targeted therapies. However, only 1 out 15 combinations in the list of synergistic 2-agent combinations included at least one monoclonal antibody (Table 1, rituximab+alpha-interferon). Although a small sample size, requiring future validation, these data support a lack of significant enrichment of synergy by the addition of a monoclonal antibody relative to other agent combinations (*p*-value = 0.99, Fischer’s exact test). This observation may also indicate that synergy is as common between chemotherapeutic agents as between a chemotherapeutic agent and a monoclonal antibody.

### Quantifying agent interactions using a 2-agent approximation

**Figure 3:**
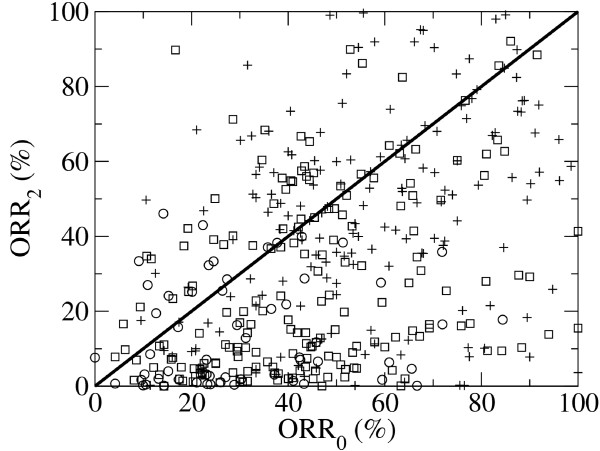
**Performance of the 2**-**agents approximation.** Scatter plot of the empirically estimated ORR (ORR_0_) as a function the leave-one-out crossvalidation prediction based on the 2-agent approximation (ORR_2_). Circles represent combinations using one agent, squares combinations using two agents and pluses combinations using three or more agents.

Understanding that the analysis assessing clinical synergy is limited by the availability of clinical trials testing each agent as a single agent and the two agents in combination, we performed a 2-agent approximation. A new agent is often added to an existing regimen that already includes two or more agents, without testing the new agent in combination with each agent within the existing regimen. Therefore, we estimated the response rate of a combination of two agents from a collection of trials where these agents appeared as part of a combination with more than two agents. We developed a model for the ORR as a function of parameters characterizing the single agent and 2-agent responses (see Methods). A leave-one-out-cross-validation scheme was used to evaluate the performance of the 2-agents approximation. For each combination, the empirically estimated ORR was removed from the dataset and then estimated using the 2-agent approximation. Figure 3 shows a scatter plot of the mean ORR as predicted by the 2-agent approximation (ORR_2_) as a function of the mean ORR empirical estimates (ORR_0_). We noticed that in most cases the predicted ORRs were lower than the empirical estimates, as can be deduced from the higher density of points in the lower-right triangle in Figure 3. Indeed, in 77, 76 and 69% of the combinations with 1, 2 and 3 or more agents, respectively, the predicted ORR was lower than the empirical estimate (ORR_2_<ORR_0_). Therefore, the 2-agent approximation can be used to estimate a lower bound to the ORR. More precisely, with about 75% confidence, the actual ORR is higher than what predicted by the 2-agent approximation. In practical terms this means that when the 2-agent approximation predicts a high ORR, with about 75% confidence we should expect as high or higher responses rates. On the other hand, if the 2-agent approximation predicts a low ORR, the prediction become less informative, since likely the ORR will actually be higher. Using the 2-agent approximation we estimated the ORR of all 2-agent combinations, provided that the two agents appeared together in at least one trial within our dataset (Additional file 4). We noted again that these predictions were likely underestimating the actual ORR. We also noted that these predictions were subject to the same biases discussed above. If an agent has been tested in a specific cancer subtype, then the predicted ORRs for combinations using that agent are expected to be valid within that subtype.

## Conclusions

Statistical and systems biology methodologies have been proposed for the rational discovery of combinatorial therapies [2]. Our ability to test the performance of these methodologies is limited, however, by the availability of a catalog of synergistic drug combinations. Similarly, a catalog of drugs with antagonistic interactions could help to design statistical and systems biology methodologies to anticipate adverse drug interactions [8]. We propose the pooled analysis of Phase II clinical trials as a methodology to develop a catalog of synergistic and antagonistic drug combinations. This catalog can be used as a gold standard for testing methodologies attempting to infer the interactions between drugs in the treatment of cancer. Here we provide a preliminary version of this catalog, derived from the analysis of 1,163 clinical trials and 53,745 subjects. The synergistic 2-agent combinations are reported in Table 1 and the antagonistic two-agent combinations in Table 2.

The pooled analysis of this data supports the hypothesis that the ORR increases with increasing the number of drugs used in combination, from 25% for single agents to about 85% for five drugs combinations. In most cases, this increase is explained by the additive effect of the drugs in the combination. However, for certain combinations, the ORRs are significantly greater than what would be expected from the additive effects of the two drugs, which represent our predicted synergistic drug combinations. On the other hand, there were also drug combinations with ORRs significantly below what is expected from their additive effects, which represent antagonistic drug combinations.

To evaluate the impact of targeted therapies, we focused on monoclonal antibodies. We recognize that there are many different and emerging targeted therapies such as kinase inhibitors. However, monoclonal antibodies were the only class present in a sufficient number to conduct the analysis. We observe a significant 8% increase in the average ORR of trials using monoclonal antibodies, indicating that these targeted therapies are indeed improving the response rates. However, among the reported synergistic combinations, there was not a significant enrichment of combinations using monoclonal antibodies. Based on this evidence, we conclude that synergy is as common between chemotherapeutic agents as between a chemotherapeutic agent and a monoclonal antibody. These methodologies will be more important as ongoing studies are now assessing more combinations of targeted agents with standard chemotherapy as well as combinations of targeted agents.

Using a 2-agent approximation we estimated the ORR of all combinations of two agents, provided the two agents were tested together, alone or in combination with other agents, in at least one clinical trial. A cross-validation analysis revealed that this 2-agent approximation provides a good lower bound estimate of the ORR. In practical terms, this means that the combinations with predicted high ORRs are quite likely to manifest high ORRs, but if the ORR is predicted to be low, then the prediction is not informative. The 2-agent approximation predictions, reported in the Additional file 4, can be used as a reference to prioritize further testing of combination therapy for cancer treatment.

The *in vitro* definition of synergy based on growth inhibition assays [9] does not necessarily predicts clinical synergy, because of drug metabolism or other factors. For example, an *in vitro* study using breast cancer cell lines, it was reported that the combination docetaxel+doxorubicin was antagonistic, while the combination docetaxel+epirubicin was synergistic [11]. In contrast, we found that the combination docetaxel+doxurubicin was synergistic based on the Phase II trials clinical data of breast cancer, while the combination of docetaxel+epirubicin was antagonistic, albeit in lung cancer. This example emphasizes that *in vitro* evidence for synergy, while potentially informative, should not be the gold standard, underscoring the need for gold standards of synergy in the clinical context.

In a more general perspective, this analysis opens the question of whether we should test both new treatments and the standard of care on each trial, or whether we should test only the new treatments in new trials and compare the results against the gold standard library. This may be particularly relevant to efforts to improve trial efficiency in order to test multiple targeted new agents in combination. Clearly, given the ORR of a new treatment, we can provide estimates of clinical benefit and synergy using our methodology. Looking ahead, the statistical methodology developed in this work can be further tested and validated with expanded phase II clinical trial data sets, more specific data sets, and in prospective predictions of new combinations moving into phase II studies.

## Competing interests

The authors declare no conflicts of interest.

## Authors’ contributions

AV and RSD designed the study, WK and AV performed the analysis of the data, WK, RSD and AV wrote the manuscript. All authors read and approved the final manuscript.

## Pre-publication history

The pre-publication history for this paper can be accessed here:

http://www.biomedcentral.com/1471-2288/13/77/prepub

## Supplementary Material

Additional file 1 Dataset of clinical trials analyzed in this work. Click here for file

Additional file 2 Detailed description of the statistical methods used in this work. Click here for file

Additional file 3 Agent combinations manifesting evidence of more than one class of response rates, depending on cancer subtype or other factors. Click here for file

Additional file 4 **Predicted ORRs based on the 2-agent approximation (ORR**_**2**_**).** The combinations were ranked in decreasing order or their predicted median ORR. When available, the empirically estimated ORR (ORR_0_) is reported as well. Click here for file

## References

[B1] Kummar S Chen HX Wright J Holbeck S Millin MD Tomaszewski J Zweibel J Collins J Doroshow JH Utilizing targeted cancer therapeutic agents in combination: novel approaches and urgent requirements Nat Rev Drug Discov 2010 9 11 843 856 10.1038/nrd3216 21031001

[B2] Feala JD Cortes J Duxbury PM Piermarocchi C McCulloch AD Paternostro G Systems approaches and algorithms for discovery of combinatorial therapies Wiley Interdiscip Rev Syst Biol Med 2010 2 2 181 193 10.1002/wsbm.51 20836021

[B3] Maitland ML Hudoba C Snider KL Ratain MJ Analysis of the yield of phase II combination therapy trials in medical oncology Clin Cancer Res 2010 16 21 5296 5302 10.1158/1078-0432.CCR-10-0669 20837695 PMC2970723

[B4] Belfiglio M Fanizza C Tinari N Ficorella C Iacobelli S Natoli C Meta-analysis of phase III trials of docetaxel alone or in combination with chemotherapy in metastatic breast cancer J Cancer Res Clin Oncol 2012 138 2 221 229 10.1007/s00432-011-1091-0 22095437 PMC3258394

[B5] Rossari JR Metzger-Filho O Paesmans M Saini KS Gennari A De Azambuja E Piccart-Gebhart M Bevacizumab and breast cancer: a meta-analysis of first-line phase III studies and a critical reappraisal of available evidence J Oncol 2012 2012 417673 23008712 10.1155/2012/417673 PMC3447373

[B6] Wu X Jin Y Cui IH Xu Z Zhang Y Zhang X Tang C Gong Y Chen J Addition of vandetanib to chemotherapy in advanced solid cancers: a meta-analysis Anticancer Drugs 2012 23 7 731 738 10.1097/CAD.0b013e32835514f4 22700001

[B7] Gao G Chu H Zhao L Gui T Xu Q Shi J A meta-analysis of paclitaxel-based chemotherapies administered once every week compared with once every 3 weeks first-line treatment of advanced non-small-cell lung cancer Lung Cancer 2012 76 3 380 386 10.1016/j.lungcan.2011.12.001 22226626

[B8] Scripture CD Figg WD Drug interactions in cancer therapy Nat Rev Cancer 2006 6 7 546 558 10.1038/nrc1887 16794637

[B9] Chou TC Theoretical basis, experimental design, and computerized simulation of synergism and antagonism in drug combination studies Pharmacol Rev 2006 58 3 621 681 10.1124/pr.58.3.10 16968952

[B10] Wilcox RR Introduction to robust estimation and hypothesis testing 2005 2 Amsterdam, Boston: Elsevier/Academic Press

[B11] Budman DR Calabro A In vitro search for synergy and antagonism: evaluation of docetaxel combinations in breast cancer cell lines Breast Cancer Res Treat 2002 74 1 41 46 10.1023/A:1016070230538 12150451

