# Supplementary material for: Inference of synergy/antagonism between anticancer drugs from the pooled analysis of clinical trials
Source: BMC Med Res Methodol. 2013 Jun 12;13:77. doi: 10.1186/1471-2288-13-77 (PMC3694030; doi:10.1186/1471-2288-13-77)
Supplement: Additional file 2 — Detailed description of the statistical methods used in this work. [file 1471-2288-13-77-S2.pdf]

Optimization of Clinical Trial Design for Combinatorial Therapies using Statistical Methodologies: Supplementary Methods 1

Wenfeng Kang[1,2], Robert S. DiPaola[3] and Alexei Vazquez[1,2]

Department of Radiation Oncology[1], Center for Systems Biology[2], and Division of Medical Oncology[3], The Cancer Institute of New Jersey and University of Medicine and Dentistry of New Jersey - Robert Wood-Johnson Medical School, New Brunswick, NJ 08901, USA

I. INTRODUCTION

In this supplementary methods we provide a more detailed description of the statistical methods utilized in the main manuscript.

II. ONE COMBINATION ONE TRIAL

In this section we discuss the simplest case of one agent combination tested in one trial of sample size N. The outcome is the number of patients n that responded to treatment. Under the assumption that all patients are equivalent the probability to obtain n responses after treating N patients is given by the binomial distribution

$$P(n|p,N) = \binom{N}{n} p^n (1-p)^{N-n} \tag{1}$$

where p is the probability that a patient responds to treatment. p is an unknown parameter that we will estimate from the available data. To do so we use the Bayes theorem

$$P(p|n,N)P(n,N) = P(n|p,N)P(p,N) \tag{2}$$

that reads, the probability of the model parameter p given the data times the probability of the data equals the probability of the data given the model parameter p times the probability of the model parameter. $P(p,N)$ is called the prior distribution and $P(p|n,N)$ the posterior distribution. In general we can assume that p and N are independent and, therefore, $P(p,N) = P(p)P(N)$ and (2) can be rewritten as

$$P(p|n,N) = \frac{1}{Z} P(n|p,N)P(p) \tag{3}$$

where $Z = P(n,N)/P(N)$. $Z(n,N)$ can also be derived from the normalization of $P(p|n,N)$: $\int_0^1 dpP(p|,n,N) = 1$, obtaining

$$Z(n,N) = \int_0^1 dpP(n|p,N)P(p) \tag{4}$$

The prior distribution of p for the binomial model is a beta distribution (2; 3)

$$P(p) = Be(p;\tilde{\alpha},\tilde{\beta}) = \frac{1}{B(\tilde{\alpha},\tilde{\beta})} p^{\tilde{\alpha}-1}(1-p)^{\tilde{\beta}-1} \tag{5}$$

where $Be(p;\tilde{\alpha},\tilde{\beta})$ denotes the beta distribution, $B(\tilde{\alpha},\tilde{\beta})$ denotes the beta function, and $\tilde{\alpha}$ and $\tilde{\beta}$ are called hyperparameters. Using symmetry arguments it can be shown that the correct choice of hyperparameters is $\tilde{\alpha} \ll 1$ and $\tilde{\beta} \ll 1$ (1).

From (3) and (5) we obtain that the posterior distribution also follows a beta distribution as well

$$P(p|n,N) = Be(p;\alpha,\beta) \tag{6}$$

where

$$\alpha = \tilde{\alpha} + n \approx n \tag{7}$$

$$\alpha = \tilde{\beta} + N - n \approx N - n \tag{8}$$

where the approximations are obtained after impossing that $\tilde{\alpha} \ll 1$ and $\tilde{\beta} \ll 1$. From the posterior distribution we can calculate different quantities. For example the posterior estimate of the mean of p

$$\text{mean}(p) = \frac{\alpha}{\alpha + \beta} \approx \frac{n}{N} \tag{9}$$

III. ONE COMBINATION MULTIPLE TRIALS

In this section we discuss the case of one agent combination that has been tested in one or more trials of sample sizes N_1, N_2, ... N_T, where T is the number of trials. The outcome is the number of patients n_i that responded to treatment in trial $i = 1, 2, \ldots, T$. In practice the clinical trials may have been conducted using different doses and/or treatment schedules and in different cancer subtypes. Thefore, the assumption that all patients are equivalent may not represent the real scenario. To deal with this case we assume that within each trial all patients are equivalent, but different trials may be characterized by different response rates. Specifically, we assume there are G groups of trials, each characterized by its one probability of response p_k, $k = 1, \ldots, G$ and each trial belongs to one of these groups. We denote by g_i the group to which trial i belongs, $i = 1, \ldots, T$. We note G and g_i are unknown parameters that will be estimated from the data. The probability to observe the data given this model is

$$P(n|p, g, N) = \prod_{i=1}^{T} \binom{N_i}{n_i} p_{g_i}^{n_i} \left(1 - p_{g_i}\right)^{N_i - n_i} \tag{10}$$

Again, using Bayes theorem we write

$$P(p, g|n, N) P(n, N) = P(n|p, g, N) P(p, g, N) \tag{11}$$

Assuming that the response rates, trial group and sample sizes are independent we obtain $P(p, g, N) = P(p)P(g)P(N)$. The prior distribution of the response rates is, as explained above, given by a beta distribution, now across multiple groups

$$P(p) = \prod_{k=1}^{G} Be(p_k; \tilde{\alpha}_k, \tilde{\beta}_k) \tag{12}$$

The prior distribution of g can be generated assuming a multinomial model (2), where a trial belong to group k with probability π_k, resulting in

$$P(g) = P(g|\pi) P(\pi) \tag{13}$$

where

$$P(g|\pi) = \prod_{i=1}^{T} \pi_{g_i} \tag{14}$$

and $P(\pi)$ is the prior distribution of π. The prior distribution of the multinomial model is the Dirichlet distribution (2), the generalization of the beta distribution,

$$P(\pi) = D(\pi; \tilde{\gamma}) = \frac{1}{B(\tilde{\gamma})} \prod_{k=1}^{G} \pi_k^{\tilde{\gamma}_k} \tag{15}$$

where

$$B(\tilde{\gamma}) = \frac{\prod_{k=1}^{G} \Gamma(\tilde{\gamma}_k)}{\Gamma\left(\sum_{k=1}^{G} \tilde{\gamma}_k\right)} \tag{16}$$

is the generalized beta function. Once again, using symmetry arguments it can be shown that the correct choice if hyperparameters is $\tilde{\gamma} \ll 1$ (1). Putting all together, from equations (10)-(15) we obtain the prior and posterior distributions

$$P(p, g, \pi) = \prod_{k=1}^{G} Be(p_k; \tilde{\alpha}_k, \tilde{\beta}_k) \prod_{i=1}^{T} \pi_{g_i} D(\pi; \tilde{\gamma}) \tag{17}$$

$$P(p, g, \pi | n, N) = \frac{1}{Z} \prod_{k=1}^{G} Be(p_k; \alpha_k, \beta_k) D(\pi; \gamma) \frac{B(\gamma)}{B(\tilde{\gamma})} \prod_{k=1}^{G} \frac{B(\alpha_k, \beta_k)}{B(\tilde{\alpha}, \tilde{\beta})} \tag{18}$$

where

$$\alpha_k = \tilde{\alpha}_k + \sum_{i=1,\ldots,T|g_i=k} n_i \tag{19}$$

$$\beta_k = \tilde{\beta}_k + \sum_{i=1,\ldots,T|g_i=k} (N_i - n_i) \tag{20}$$

$$\gamma_k = \tilde{\gamma}_k + \sum_{i=1,\ldots,T|g_i=k} 1 \tag{21}$$

We can integrate (18) over the p and π variables to obtain the marginal posterior distribution for g

$$P(g|n, N) = \frac{1}{Z} \frac{B(\gamma)}{B(\tilde{\gamma})} \prod_{k=1}^{G} \frac{B(\alpha_k, \beta_k)}{B(\tilde{\alpha}, \tilde{\beta})} \tag{22}$$

Using equation (22) we can discriminate between different clusterings of the trials, including the case were all trials belong to one group, and select the one with highest probability. The hyperparameters $\tilde{\alpha}$, $\tilde{\beta}$ and $\tilde{\gamma}$ should be choosen as small as possible, taking into account that too small values may compromise the numerical accuracy when computing the Γ function. We used $\tilde{\alpha} = \tilde{\beta} = \tilde{\gamma} = 1 \times 10^{-2}$. For no so large T and G, we can evaluate equation (22) for all possible clusterings of the T trials in K groups and select the group with largest likelihood $P(g|n, N)$.

As a case study, we analyzed the case when there are two different trials with response probability p_1 and p_2 and sample sizes N_1 and N_2. At each simulation of the trials, we generated values of n_1 and n_2 from the binomial distributions $p(n_1|p_1, N_1)$ and $p(n_2|p_2, N_2)$. Then, using equation(22), we determined if the trials fall into one or two different groups. For the case $N_1 = N_2$ and $p_2 = 1 - p_1$ ($p_2 - p_1 = 1 - 2p_1$), the Supplementary Figure 1 shows the fraction of times the variational method predicted that the two trials are statistically different in 1,000 trial simulations. When $p_2 - p_1 > 0.3$, in most cases the variational method correctly predicts that n_1 and n_2 are generated by differnt distributions. In contrast, when $p_2 - p_1 < 0.1$, in most cases the variational method fail to detect that n_1 and n_2 are generated from different distributions. This is, however, expected since for $p_2 - p_1 < 0.1$ the distributions

of n_1 and n_2 are quite close to each other. The transition between these two extreme regimes is smoother or sharper depending on the sample sizes, being sharper the larger the sample sizes.

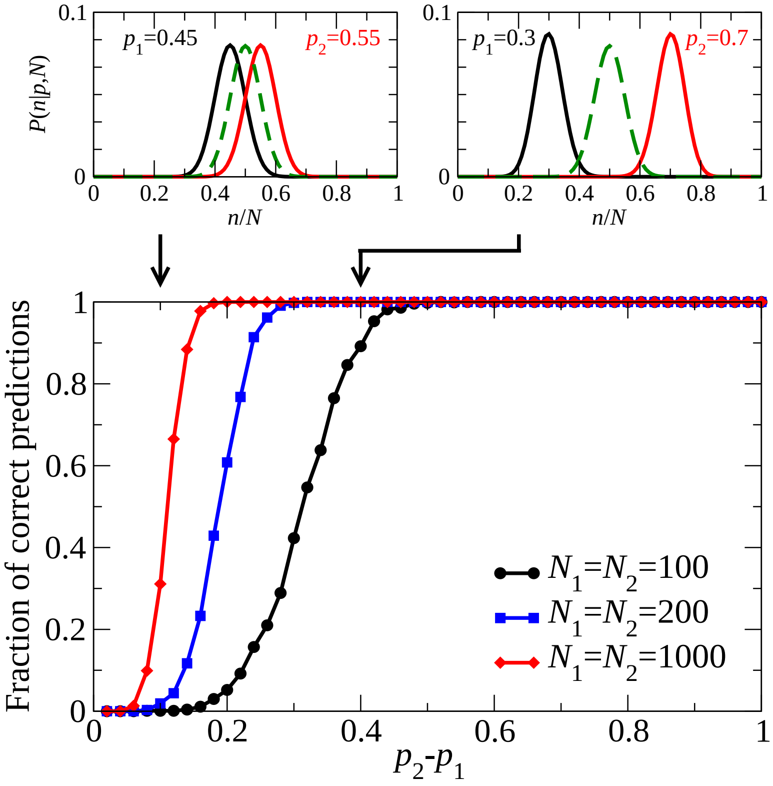

Supplementary Figure 1: Performance of the variational method for the case $N_1 = N_2$ and $p_2 = 1 - p_1$. The upper panels show the distributions $P(n|p_1, 100)$ (black), $P(n|p_2, 100)$ (red) and $P(n|, 0.5, 100)$ (green-dashed) for $p_1 = 0.45$ and $p_2 = 0.55$ (left) and $p_1 = 0.3$ and $p_2 = 0.7$ (right).

IV. 2-AGENTS APPROXIMATION

In this section we describe the numerical implementation of the 2-agent approximation. The 2-agent approximation is given by

$$p_c = \sum_{i=1}^{N_a} s_{ci} h_i + \sum_{i=1}^{N_a-1} \sum_{j=i+1}^{N_a} s_{ci} s_{cj} J_{ij} \ , \quad 1 \leq c \leq C \tag{23}$$

where C is the number of combinations, N_a is the number of agents, s_{ci} is the agent to combination matrix ($s_{ci} = 1$ if agent i is used in combination c and $s_{ci} = 0$ otherwise), the h_i quantifies the response of single agents and the parameters J_{ij} the interactions between agents i and j. We can write (23) in a more compact form after introducing the following notation. We create a list with all the hs and Js parameters, arranged such that the element k contains the parameter h_k for $1 \leq k \leq N_a$ and the Js for $N_a + 1 \leq k > N_p$, where N_p is the total number of hs and Js. In the most general scenario there are $N_a(N_a - 1)/2$ of Js, one for each pair of agents, and $N_p = N_a + N_a(N_a - 1)/2$. However, the 2-agents approximation can only constraint a give J_{ij} if there is at least one combination containing agents i and j. The Js values that cannot be constrained are removed. We denote by N_J the number of Js that are not removed, resulting in $N_p = N_a + N_J$. We will also denonte by i_k and j_k the i and j associated with the k-th J_{ij} element, $N_a + 1 \leq k \leq N_p$, in the parameters list. Using this notation we we can write (23) as

$$\sum_{k=1}^{N_p} A_{ck} x_k = p_c \ , \quad 1 \leq c \leq C \tag{24}$$

where

$$A = \begin{cases} s_{ck} \ , & 1 \le c \le C \quad 1 \le k \le N_a \\ s_{ci_k} s_{cj_k} \ , & 1 \le c \le C \quad N_a + 1 \le k \le N_p \end{cases} \tag{25}$$

$$x = \begin{cases} h_k \ , & 1 \le k \le N_a \\ J_{i_k,j_k} \ , & N_a + 1 \le k \le N_p \end{cases} \tag{26}$$

The system of equations (24) has C equations and N_p variables. In the problem considered within the main manuscript $N_p > C$. Taking into account that we do not know the precise values of p_c but its distribution, we double the number equations as follows

$$\sum_{k=1}^{N_p} A_{ck} x_k = b_c \ , \quad 1 \le c \le 2C \tag{27}$$

$$A = \begin{cases} s_{ck} \ , & 1 \le c \le C \ , & 1 \le k \le N_a \\ s_{ci_k} s_{cj_k} \ , & 1 \le c \le C \ , & N_a + 1 \le k \le N_p \\ s_{c-C,k} \ , & C + 1 \le c \le 2C \ , & 1 \le k \le N_a \\ s_{c-C,i_k} s_{c-C,j_k} \ , & C + 1 \le c \le 2C \ , & N_a + 1 \le k \le N_p \end{cases} \tag{28}$$

$$b = \begin{cases} \text{random number from } Be(\alpha_c, \beta_c) \ , & 1 \le c \le C \\ \text{random number from } Be(\alpha_{c-C}, \beta_{c-C}) \ , & C + 1 \le c \le 2C \end{cases} \tag{29}$$

In this way we obtain a system of $2C$ equations and N_p variables. In the problem considered in the main manuscript $2C > N_p$, resulting in more equations than variables. Finally, we take into account that the response rates take values between 0 and 1, imposing constraints on the hs and Js. In particular, since the hs represent the response rates for single agent combinations, they take values between 0 and 1, i.e. $0 \le x_k \le 1$ for $1 \le k \le N_a$. Similarly, for every combination of two agents we have $0 \le h_{i_k} + h_{j_k} + J_{i_k,j_k} \le 1$, $N_a + 1 \le k \le N_p$. This constraint can be taken into account introducing the auxiliary equations $h_{i_k} + h_{j_k} + J_{i_k,j_k} - x_{N_J+k} = 0$ with the auxiliary variables x_{N_J+k} satisfying $0 \le x_{N_J+k} \le 1$, $N_a+1 \le k \le N_p$. We also note that $0 \le h_{i_k} + h_{j_k} + J_{i_k,j_k} \le 1$ implies that $-2 \le J_{i_k,j_k} \le 1$. After adding this constraint we augments our systems of equations to

$$\sum_{k=1}^{N_v} A_{ck} x_k = b_c \ , \quad 1 \le c \le 2C + N_J \tag{30}$$

$$l_k \le x_k \le 1 \ , \quad 1 \le k \le N_v \tag{31}$$

where $N_v = N_p + N_J$ and

$$A = \begin{cases} s_{ck} \ , & 1 \le c \le C \ , & 1 \le k \le N_a \\ s_{ci_k} s_{cj_k} \ , & 1 \le c \le C \ , & N_a + 1 \le k \le N_p - N_a \\ 0 \ , & 1 \le c \le C \ , & N_p + 1 \le k \le N_v \\ s_{c-C,k} \ , & C + 1 \le c \le 2C \ , & 1 \le k \le N_a \\ s_{c-C,i_k} s_{c-C,j_k} \ , & C + 1 \le c \le 2C \ , & N_a + 1 \le k \le N_p - N_a \\ 0 \ , & C + 1 \le c \le 2C \ , & N_p + 1 \le k \le N_v \\ 1 - (1 - \delta_{k,i_k})(1 - \delta_{k,j_k}) \ , & 2C + 1 \le c \le 2C + N_J \ , & 1 \le k \le N_a \\ \delta_{c-2C,k} \ , & 2C + 1 \le c \le 2C + N_J \ , & N_a + 1 \le k \le N_J \\ -1 \ , & 2C + 1 \le c \le 2C + N_J \ , & N_p + 1 \le k \le N_v \end{cases} \tag{32}$$

$$b = \begin{cases} \text{random number from } Be(\alpha_c, \beta_c) \ , & 1 \le c \le C \\ \text{random number from } Be(\alpha_{c-C}, \beta_{c-C}) \ , & C + 1 \le c \le 2C \\ 0 \ , & 2C + 1 \le c \le 2C + N_J \end{cases} \tag{33}$$

$$l = \begin{cases} 0 \ , & 1 \le k \le N_a \\ -2 \ , & N_a + 1 \le k \le N_p \\ 0 \ , & N_p + 1 \le k \le N_v \end{cases} \tag{34}$$

where $\delta_{ij} = 1$ when $i = j$ and 0 otherwise.

A. Numerical solution using Matlab

We solved the problem (30) using the Matlab function `lsqlin`. To this end, at each simulation, we generated the random vectors (33) and obtained the least-squares solution to (30) under the lower/upper bound constraints (31), and consequently the associated hs and Js. The simulation was repeated 1,000 times to estimate the distribution of the hs and Js parameters.

B. The case of different response rates for the same combination

References

[1] Vazquez A (2009) Finding hypergraph communities: a Bayesian approach and variational solution, J. Stat. Mech. P07006.
[2] Blei D M, Ng A Y and Jordan M I, Latent Dirichlet allocation (2003). J Mach Learn. Res 3 993.
[3] MacKay D J C, 2003 Information Theory, Inference, and Learning Algorithms (Cambridge: Cambridge University Press).
[4] Beal M J, Variational algorithms for approximate Bayesian inference, 2003 PhD Thesis Gatsby Computational Neuroscience Unit, University College London.
